# Ventricular Arrhythmias After Primary Percutaneous Coronary Intervention for STEMI

**DOI:** 10.1001/jamanetworkopen.2024.10288

**Published:** 2024-05-08

**Authors:** Jennifer A. Rymer, Zachary K. Wegermann, Tracy Y. Wang, Shuang Li, Nathaniel R. Smilowitz, B. Hadley Wilson, Hani Jneid, Jacqueline E. Tamis-Holland

**Affiliations:** 1 Department of Cardiology, Duke University Hospital, Durham, North Carolina; 2 Patient-Centered Outcomes Research Institute, Washington, DC; 3 Duke Clinical Research Institute, Durham, North Carolina; 4 Department of Medicine, NYU Langone Medical Center, New York, New York; 5 Department of Cardiology, Atrium Health Sanger Heart & Vascular Institute, Charlotte, North Carolina; 6 Department of Medicine, University of Texas Medical Branch, Galveston; 7 Department of Cardiology, Heart, Vascular & Thoracic Institute, Cleveland Clinic, Cleveland, Ohio

## Abstract

**Importance:**

Currently, mortality risk for patients with ST-segment elevation myocardial infarction (STEMI) treated with primary percutaneous coronary intervention (PCI) with an uncomplicated postprocedure course is low. Less is known regarding the risk of in-hospital ventricular tachycardia (VT) and ventricular fibrillation (VF).

**Objective:**

To evaluate the risk of late VT and VF after primary PCI for STEMI.

**Design, Setting, and Participants:**

This cohort study included adults aged 18 years or older with STEMI treated with primary PCI between January 1, 2015, and December 31, 2018, identified in the US National Cardiovascular Data Registry Chest Pain–MI Registry. Data were analyzed from April to December 2020.

**Main Outcomes and Measures:**

Multivariable logistic regression was used to evaluate the risk of late VT (≥7 beat run of VT during STEMI hospitalization ≥1 day after PCI) or VF (any episode of VF≥1 day after PCI) associated with cardiac arrest and associations between late VT or VF and in-hospital mortality in the overall cohort and a cohort with uncomplicated STEMI without prior myocardial infarction or heart failure, systolic blood pressure less than 90 mm Hg, cardiogenic shock, cardiac arrest, reinfarction, or left ventricular ejection fraction (LVEF) less than 40%.

**Results:**

A total of 174 126 eligible patients with STEMI were treated with primary PCI at 814 sites in the study; 15 460 (8.9%) had VT or VF after primary PCI, and 4156 (2.4%) had late VT or VF. Among the eligible patients, 99 905 (57.4%) at 807 sites had uncomplicated STEMI. The median age for patients with late VT or VF overall was 63 years (IQR, 55-73 years), and 75.5% were men; the median age for patients with late VT or VF with uncomplicated STEMI was 60 years (IQR, 53-69 years), and 77.7% were men. The median length of stay was 3 days (IQR, 2-7 days) for the overall cohort with late VT or VF and 3 days (IQR, 2-4 days) for the cohort with uncomplicated STEMI with late VT or VF. The risk of late VT or VF was 2.4% (overall) and 1.7% (uncomplicated STEMI). Late VT or VF with cardiac arrest occurred in 674 patients overall (0.4%) and in 117 with uncomplicated STEMI (0.1%). LVEF was the most significant factor associated with late VT or VF with cardiac arrest (adjusted odds ratio [AOR] for every 5-unit decrease ≤40%: 1.67; 95% CI, 1.54-1.85). Late VT or VF events were associated with increased odds of in-hospital mortality in the overall cohort (AOR, 6.40; 95% CI, 5.63-7.29) and the cohort with uncomplicated STEMI (AOR, 8.74; 95% CI, 6.53-11.70).

**Conclusions and Relevance:**

In this study, a small proportion of patients with STEMI treated with primary PCI had late VT or VF. However, late VT or VF with cardiac arrest was rare, particularly in the cohort with uncomplicated STEMI. This information may be useful when determining the optimal timing for hospital discharge after STEMI.

## Key Points

**Question:**

What is the risk of late ventricular tachycardia (VT) and ventricular fibrillation (VF) after primary percutaneous coronary intervention (PCI) for patients with ST-segment elevation myocardial infarction (STEMI)?

**Findings:**

In this cohort study of 174 126 patients with STEMI, 8.9% of patients had VT or VF after primary PCI. However, VT or VF occurring 1 or more days after primary PCI and associated with cardiac arrest was rare, occurring in 0.4% of all patients and 0.1% of patients with uncomplicated STEMI.

**Meaning:**

This information may be useful when determining the optimal timing for hospital discharge after STEMI.

## Teaser

This cohort study examines the risk of ventricular tachycardia or ventricular fibrillation 1 or more days after primary percutaneous coronary intervention for ST-segment elevation myocardial infarction (STEMI).

## Introduction

Ventricular tachycardia (VT) and ventricular fibrillation (VF) are life-threatening complications of acute ST-segment elevation myocardial infarction (STEMI). Prior data suggest VT occurs in 5% to 10% of patients with acute STEMI, with two-thirds of cases occurring prior to reperfusion and 90% within the first 48 hours after infarction.^1,2,3,4,5^ Late VT or VF, occurring more than 24 hours after reperfusion, is thought to be rare in the overall population with STEMI, with studies suggesting an incidence of 0.6% to 1.7% in patients with STEMI treated with primary percutaneous coronary intervention (PCI).^1,2^ However, limited data exist in the contemporary era regarding the incidence and timing of VT or VF in patients with STEMI, with most data derived from noncontemporary clinical trials and small cohort studies.^1,2,4,5,6,7,8^

Given advancements in reperfusion therapy with primary PCI and systems of care to improve access to timely reperfusion,^9,10^ a contemporary evaluation of the risk, predictors of, and outcomes associated with VT or VF is needed. The lack of contemporary evidence on VT or VF after STEMI makes it difficult to determine the optimal duration of in-hospital telemetry monitoring following STEMI and which patients may be eligible for early discharge, which is reflected in the lack of clear guidance in current guidelines.^11,12^ As such, a contemporary analysis of the risk of VT or VF following STEMI treated by primary PCI is needed with a focus on a subpopulation of patients with uncomplicated STEMI who are likely to be at low risk for VT or VF events based on presenting risk factors.

In this analysis, we sought to (1) examine the risk and timing of VT or VF events in patients with STEMI treated with primary PCI, (2) measure in-hospital mortality associated with late VT or VF, and (3) identify factors associated with late VT or VF events in both the overall population of patients presenting with STEMI treated with primary PCI and a subpopulation with uncomplicated STEMI.

## Methods

### Data Source

Data for this cohort study were obtained from the National Cardiovascular Data Registry (NCDR) Chest Pain–MI Registry, a voluntary registry of 1295 US sites that collects data on consecutive patients admitted with acute myocardial infarction (MI) at participating centers across the US.^13^ Patient characteristics, including medical history, clinical presentation details, in-hospital treatments, and in-hospital outcomes, are abstracted by trained abstractors at participating sites via medical record review. Regular audits and data quality checks are performed to ensure data accuracy within the registry. The Duke University Health System institutional review board approved the study with a waiver of informed consent because data were from a national retrospective registry. This analysis followed the Strengthening the Reporting of Observational Studies in Epidemiology (STROBE) reporting guideline.

### Study Population

The study population included unique, consecutive patients included in the Chest Pain–MI Registry between January 1, 2015, and December 31, 2018. We also specified a cohort of patients with uncomplicated STEMI treated with primary PCI, defined as patients without reinfarction on the day of PCI, systolic blood pressure less than 90 mm Hg at admission, cardiogenic shock at admission or the same day as PCI, cardiac arrest or need for intubation before PCI, prior MI, prior heart failure, heart failure at first medical contact, or a left ventricular ejection fraction (LVEF) less than 40%. Patients presenting without STEMI and patients with STEMI who did not undergo primary PCI were excluded. After excluding patients with cardiac arrest at first medical contact, patients who transferred out within the first 48 hours of admission, patients with missing in-hospital VT or VF data, and patients with missing dates for primary PCI, VT, or VF, a final study cohort of patients from 814 sites was available for analysis. Among these patients, those with VT or VF on the day of or before primary PCI were excluded.

Race and ethnicity data were ascertained by self-report and were included to evaluate racial and ethnic differences in care. Categories were Asian, Black, Hispanic, White, and other (included American Indian or Alaskan Native, Native Hawaiian or Pacific Islander, and groups with too few participants to characterize further).

### Study Outcomes

We examined the risk and timing of the first episode of VT or VF, the factors associated with development of late VT or VF, and the association between late VT or VF and in-hospital mortality. The follow-up period was the in-hospital period. Ventricular tachycardia was defined within the NCDR Chest Pain–MI Registry as a run of 7 or more beats of VT at any time during the index hospitalization regardless of hemodynamic significance. Ventricular fibrillation was defined as any episode of VF during the index hospitalization. We defined late VT or VF as any VT or VF event occurring at least 1 day after primary PCI. We also examined late VT or VF associated with cardiac arrest, defined as VT or VF occurring on the same day as in-hospital cardiac arrest. In-hospital mortality was defined within the NCDR Chest Pain–MI Registry as a patient death at any time between index hospital admission and discharge.

### Statistical Analysis

The risk of VT or VF overall, VT or VF occurring on the same day as primary PCI, VT or VF occurring at least 1 day after primary PCI, and VT or VF associated with in-hospital cardiac arrest was calculated among all patients with STEMI treated with primary PCI. The timing of the first episode of late VT or VF was presented as a distribution of days from primary PCI. Patient characteristics were compared between those with late VT or VF and those without VT or VF. Categorical variables were summarized using frequencies and percentages and compared using a χ^2^ test. Continuous variables were presented as medians and IQRs and compared using a Wilcoxon rank sum test.

In the lower-risk cohort with uncomplicated STEMI, we assessed the risk of VT or VF and compared patient characteristics between those with late VT or VF and those without VT or VF. Among these patients, those with VT or VF on the day of or before primary PCI were excluded.

To assess the association between late VT or VF and in-hospital mortality, generalized estimating equations (GEE) logistic regression models were used with adjustment for clustering within sites to compare in-hospital mortality between patients with late VT or VF and those without VT or VF after adjusting for case mix. The GEE method was implemented with a compound symmetric working correlation matrix and empirical (sandwich) SE estimates. Patient-level covariates for risk adjustment consisted of previously identified and validated covariates associated with in-hospital mortality within the registry. These included age (in years), heart failure at admission, heart rate (bpm) at presentation, systolic blood pressure (mm Hg) at presentation, cardiogenic shock at presentation, creatinine clearance (Cockcroft-Gault formula), and initial troponin ratio (a multiple of the upper limit of normal). The association between late VT or VF and in-hospital mortality was presented as an unadjusted odds ratio (OR) and a risk-adjusted OR (AOR) with 95% CIs. The association with mortality was tested in the overall primary PCI-treated population with STEMI and in the population with uncomplicated STEMI.

To identify factors associated with late VT or VF, a GEE model was developed using candidate variables selected a priori. Colinearity of candidate variables was assessed using variance inflation factors (VIFs), with large VIF measures (>5) examined closely to determine whether a variable should be dropped. No VIF measures were greater than 5 within the model. Continuous variables within the model were assessed for linearity using restricted cubic splines and the Wald χ^2^ test. If nonlinearity was found, linear spline terms with knots were used. Missingness of variables within the model was very low, and simple imputation was used to address missingness of variables within the model, with continuous variables imputed to the median value and categorical variables imputed to the most frequent group. A C-index was calculated for the developed model. A prespecified cut-point C-index of 0.75 was used to determine whether the same model should be developed in the cohort with uncomplicated STEMI. As a sensitivity analysis, we repeated the same model in the overall cohort assessing the association between candidate variables and late VT or VF associated with cardiac arrest. While the C-index met the prespecified end point, there were too few events in the low-risk cohort to develop a model.

All statistical analyses were performed from April to December 2020, using SAS, version 9.4 (SAS Institute Inc). All hypothesis tests were 2-sided, *P* < .05 was the nominal threshold for statistical significance, and nominal 95% CIs were calculated. Due to the exploratory nature of this observational research, there was no adjustment for multiple hypothesis testing for secondary and subgroup analyses.

## Results

Between January 1, 2015, and December 31, 2018, a total of 582 402 unique, consecutive patients were included in the Chest Pain-MI registry. After excluding 356 639 presenting with non-STEMI, 34 508 with STEMI who did not undergo primary PCI, 13 874 with cardiac arrest at first medical contact, 2888 who transferred out within the first 48 hours of admission, 198 with missing in-hospital VT or VF data, and 169 with missing dates for primary PCI or VT and VF, 174 126 patients from 814 sites were available for analysis. Among these patients, 11 304 with VT or VF on the day of or before primary PCI were excluded, leaving 162 822 patients. A total of 158 666 did not have VT or VF, and 4156 had late VT or VF. Among the 174 126 patients in the analytic sample, 99 905 (57.4%) had uncomplicated STEMI treated with primary PCI at 807 hospitals, and 1715 of these patients (1.7%) had late VT or VF (eFigure 1 in Supplement 1). Of the 162 822 total patients, 2.5% were Asian; 9.2%, Black; 6.7%, Hispanic; 80.2%, White; and 0.6%, other race and ethnicity. The median age of patients with late VT or VF (overall) was 63 years (IQR, 55-73 years); 75.5% were men, and 24.5% were women. The median age of patients with late VT or VF (uncomplicated STEMI) was 60 years (IQR, 53-69 years); 77.7% were men, and 22.3% were women. The median length of stay was 3 days (IQR, 2-7 days) for patients with late VT or VF overall and 3 days (IQR, 2-4 days) for the cohort with uncomplicated STEMI who had late VT or VF.

### Risk of VT or VF Events

The Figure shows the risk of VT or VF events in the overall population and the cohort with uncomplicated STEMI by the number of days after primary PCI. The risk of VT or VF in the overall primary PCI–treated population with STEMI (174 126) was 8.9% (n = 15 460), with 2.4% of patients (n = 4156) experiencing late VT or VF occurring 1 or more days after primary PCI. The majority of late VT and VF events (2619 [63.0%]) occurred on the day after primary PCI (eFigure 2 in Supplement 1). While 1537 late VT or VF events (37.0%) occurred 2 or more days after primary PCI, this represents only 0.9% of the overall population with STEMI. Among the 4156 patients with late VT or VF events, 675 (16.2%) had VT or VF associated with cardiac arrest, representing 0.4% of the overall cohort.

**Figure.**
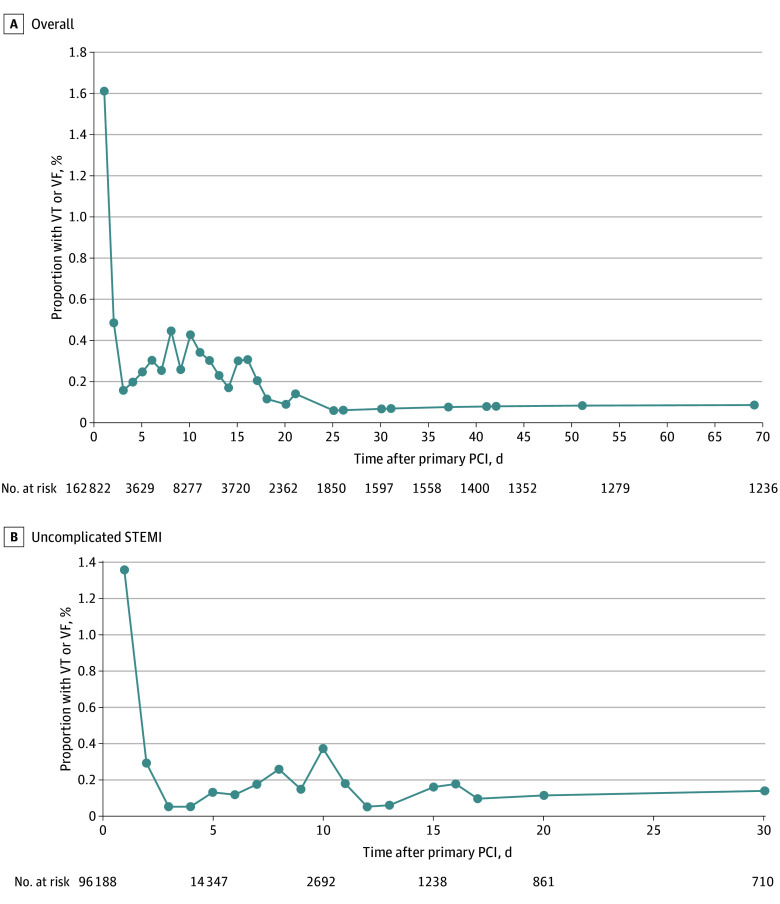
Risk of Late Ventricular Tachycardia (VT) or Ventricular Fibrillation (VF) Following Primary Percutaneous Coronary Intervention (PCI) for ST-Segment Elevation Myocardial Infarction (STEMI) The end date was discharge from the hospital. Since events were not recorded after discharge, the number of patients evaluated over time diminished; thus, the curves flatten after about 7 days.

In the predefined cohort with uncomplicated STEMI, the risk of VT or VF was 5.4% (n = 5432), with 1.7% of patients (n = 1715) experiencing late VT or VF. Among patients with late VT or VF in the cohort with uncomplicated STEMI, 1296 (75.6%) had their first VT or VF event on the day after PCI, which represents 1.3% of patients with uncomplicated STEMI. The risk of late VT or VF occurring on or after day 2 following PCI was 0.4% (419 of all patients with uncomplicated STEMI). Among patients with late VT or VF events in the cohort with uncomplicated STEMI, 117 (6.8%) had VT or VF associated with cardiac arrest, representing 0.1% of the cohort.

### Factors Associated With Late VT or VF

eTable 1 in Supplement 1 gives the characteristics of patients with late VT or VF in the overall cohort and the cohort with uncomplicated STEMI. In the overall cohort, patients with late VT or VF were older (median age, 63 years [IQR, 55-73 years] vs 61 years [IQR, 53-70 years]; *P* < .001), more likely to be White (83.2% vs 80.1%; *P* < .001), and had a higher prevalence of prior MI (17.7% vs 14.6%; *P* < .001) and prior heart failure (7.4% vs 4.0%; *P* < .001) compared with patients without VT or VF (Table 1). Despite differences in prior cardiovascular disease, there were no significant differences in baseline cardiac medications between patients with late VT or VF and those with no VT or VF. Patients with late VT or VF were more likely to arrive by ambulance (52.1% vs 48.2%; *P* < .001) and have signs of heart failure (11.0% vs 5.1%; *P* < .001) and cardiogenic shock (7.8% vs 3.6%; *P* < .001) at arrival. While the median time from symptom onset to arrival was longer among those with late VT or VF (1.68 hours [IQR, 1.00-3.18 hours] vs 1.58 hours [IQR, 0.97-3.18 hours]; *P* = .005), there were no differences between the groups in median time from arrival to catheterization (late VT or VF: 0.75 [IQR, 0.52-1.02]; no VT or VF: 0.75 [IQR, 0.50-1.03]; *P* = .81). Patients with late VT or VF were more likely to have multivessel disease (60.7% vs 54.0%; *P* < .001) and an LVEF of 50% or less (64.0% vs 44.3%; *P* < .001).

**Table 1.** Baseline Characteristics of Patients With Late VT or VF and Those Without VT or VF

Variable	Patients^a^			*P* value
	Overall (N = 162 822)	No VT or VF (n = 158 666)	Late VT or VF (n = 4156)	
Demographics				
Age, median (IQR), y	61 (53-70)	61 (53-70)	63 (55-73)	<.001
Weight, median (IQR), kg	86.0 (73.8-99.8)	86.0 (73.7-99.8)	86.0 (74.4-99.8)	.70
BMI, median (IQR)	28.7 (25.4-32.6)	28.7 (25.4-32.6)	28.3 (25.1-32.4)	<.001
Sex				
Men	115 997 (71.2)	112 863 (71.1)	3134 (75.4)	<.001
Women	46 825 (28.8)	45 803 (28.9)	1022 (24.6)	
Race and ethnicity				
Asian	4138 (2.5)	4075 (2.6)	63 (1.5)	<.001
Black	14 994 (9.2)	14 642 (9.2)	352 (8.5)	
Hispanic	10 880 (6.7)	10 651 (6.7)	229 (5.5)	
White	130 569 (80.2)	127 113 (80.1)	3456 (83.2)	
Other^b^	984 (0.6)	960 (0.6)	24 (0.6)	
Medical history				
Current or recent smoker, within 1 y	63 313 (38.9)	61 681 (38.9)	1632 (39.3)	.61
Hypertension	106 151 (65.2)	103 496 (65.2)	2655 (63.9)	.07
Dyslipidemia	84 486 (51.9)	82 342 (51.9)	2144 (51.6)	.68
Diabetes	44 444 (27.3)	43 378 (27.3)	1066 (25.7)	.02
Prior MI	23 923 (14.7)	23 187 (14.6)	736 (17.7)	<.001
Prior heart failure	6636 (4.1)	6329 (4.0)	307 (7.4)	<.001
Prior PCI	27 853 (17.1)	27 073 (17.1)	780 (18.8)	.004
Prior CABG	7693 (4.7)	7457 (4.7)	236 (5.7)	.003
Atrial fibrillation or flutter	7908 (4.9)	7593 (4.8)	315 (7.6)	<.001
Cerebrovascular disease	11 223 (6.9)	10 892 (6.9)	331 (8.0)	.006
Peripheral arterial disease	6810 (4.2)	6588 (4.2)	222 (5.3)	<.001
Receiving dialysis	1238 (0.80)	1195 (0.8)	43 (1.0)	.04
Cancer	13 087 (8.0)	12 654 (8.0)	433 (10.4)	<.001
Home medications				
Aspirin	48 385 (29.7)	47 143 (29.7)	1242 (29.9)	.82
P2Y12 inhibitors	11 598 (7.1)	11 307 (7.1)	291 (7.0)	.75
ACE inhibitor or ARB	47 714 (29.3)	46 512 (29.3)	1202 (28.9)	.57
β Blocker	37 904 (23.3)	36 887 (23.3)	1017 (24.5)	.07
Statin	46 612 (28.6)	45 404 (28.6)	1208 (29.1)	.54
Location of first evaluation				
Emergency department	120 999 (74.3)	117 931 (74.3)	3068 (73.8)	.50
Catheterization laboratory	39 154 (24.1)	38 145 (24.0)	1009 (24.3)	
Other	2488 (1.5)	2416 (1.5)	72 (1.7)	
First ECG obtained				
Before first hospital arrival	70 466 (43.3)	68 529 (43.2)	1937 (46.6)	<.001
After first hospital arrival	92 171 (56.6)	89 953 (56.7)	2218 (53.4)	
Means of transport to first facility				
Self or family	81 470 (50.0)	79 585 (50.2)	1885 (45.3)	<.001
Ambulance	78 590 (48.3)	76 423 (48.2)	2167 (52.1)	
Air	2580 (1.6)	2481 (1.6)	99 (2.4)	
Time from symptom onset to arrival, median (IQR), h	1.60 (0.97-3.18)	1.58 (0.97-3.18)	1.68 (1.00-3.18)	.005
Time from arrival to catheterization, median (IQR), h	0.75 (0.50-1.03)	0.75 (0.50-1.03)	0.75 (0.52-1.02)	.81
Signs and symptoms at presentation				
Signs of heart failure	8525 (5.2)	8067 (5.1)	458 (11.0)	<.001
Signs of cardiogenic shock	5988 (3.7)	5662 (3.6)	326 (7.8)	<.001
Heart rate at admission, median (IQR), bpm	78 (66-92)	78 (66-92)	80 (66-95)	<.001
Systolic BP at admission, median (IQR), mm Hg	146 (124-167)	146 (124-168)	140 (118-162)	<.001
Laboratory results and diagnostic data				
Diseased vessels, No.				
0	1004 (0.6)	987 (0.6)	17 (0.4)	<.001
1	73 069 (45.2)	71 460 (45.3)	1609 (38.9)	
2	49 541 (30.6)	48 220 (30.6)	1321 (31.9)	
3	38 075 (23.5)	36 883 (23.4)	1192 (28.8)	
LVEF, %				
>50	87 910 (55.2)	86 437 (55.7)	1473 (36.0)	<.001
>40 to 50	39 211 (24.6)	38 190 (24.6)	1021 (25.0)	
25 to 40	26 806 (16.8)	25 656 (16.5)	1150 (28.1)	
≤25	5443 (3.4)	4996 (3.2)	447 (10.9)	
Initial nondialysis eGFR, mL/min/1.73m^2^				
<30	3550 (2.2)	3393 (2.2)	157 (3.8)	<.001
30 to <60	34 226 (21.2)	33 145 (21.1)	1081 (26.3)	
60 to <90	76 534 (47.4)	74 633 (47.4)	1901 (46.2)	
≥90	44 878 (27.8)	43 948 (27.9)	930 (22.6)	
Initial hemoglobin level, median (IQR), g/dL	14.70 (13.40-15.80)	14.70 (13.40-15.80)	14.60 (13.20-15.70)	.001
Initial troponin level × upper limit value, median (IQR)	2.50 (0.50-33.00)	2.43 (0.50-32.50)	3.33 (0.67-55.00)	<.001

Abbreviations: ACE, angiotensin-converting enzyme; ARB, angiotensin receptor blocker; BMI, body mass index (calculated as weight in kilograms divided by height in meters squared); BP, blood pressure; CABG, coronary artery bypass graft; ECG, electrocardiogram; eGFR, estimated glomerular filtration rate; LVEF, left ventricular ejection fraction; MI, myocardial infarction; PCI, percutaneous coronary intervention; VF, ventricular fibrillation; VT, ventricular tachycardia.

SI conversion factor: To convert hemoglobin to grams per liter, multiply by 10.0.

^a^ Data are presented as number (percentage) of patients unless otherwise indicated.

^b^ Includes American Indian or Alaskan Native, Native Hawaiian and Pacific Islander, and groups with too few participants to characterize further.

In the cohort with uncomplicated STEMI, patients with late VT or VF had patterns of patient characteristics compared with those without VT or VF similar to those reported for the overall cohort, with a few notable exceptions. There was no difference in age, use of guideline-directed medications for coronary artery disease prior to admission was lower among those with late VT or VF, and patients with late VT or VF had slightly shorter median times from hospital arrival to catheterization (0.72 hours [IQR, 0.48-0.97 hours] vs 0.73 hours [IQR, 0.50-1.02 hours]; *P* = .005).

In a multivariable regression model developed to identify factors associated with late VT or VF, the most significant variable was LVEF, followed by male sex, systolic BP at admission, and cardiogenic shock at admission. Other important factors included diabetes, worsening kidney function, heart failure at admission, and off-hours presentation. Variables associated with late VT or VF are presented in Table 2. The C-index of the model was 0.663. Given that the C-index did not meet the prespecified level of 0.75 or greater, a separate model in the cohort with uncomplicated STEMI was not developed.

**Table 2.** Factors Associated With Risk of Late VT or VF Events in the Overall Cohort

Factor	AOR (95% CI)^a^	Individual *P* value	Global *P* value	χ^2^^b^
LVEF				
Per 5-unit decrease if ≤40%	1.33 (1.28-1.39)	<.001	<.001	220.80
Per 5-unit decrease if >40%	1.15 (1.12-1.18)	<.001		
Female (reference: male)	0.75 (0.69-0.81)	<.001	NA	51.59
Systolic BP on admission, per 5-unit decrease	1.02 (1.01-1.02)	<.001	NA	34.01
Cardiogenic shock at admission	1.47 (1.29-1.68)	<.001	NA	33.12
Diabetes	0.83 (0.77-0.89)	<.001	NA	27.86
Initial eCrCl, per 5-unit decrease^c^	1.01 (1.01-1.02)	<.001	NA	23.37
Heart failure at admission	1.34 (1.19-1.52)	<.001	NA	22.33
Off-hours presentation^d^	1.18 (1.09-1.27)	<.001	NA	19.10
Diseased vessels, No. (reference: 0)				
1	1.32 (0.82-2.12)	.26	.002	15.16
2	1.47 (0.90-2.38)	.12		
3	1.52 (0.94-2.47)	.09		
Initial troponin level × upper limit value				
Per 0.5-unit increase if ≤2.5 ng/mL	1.01 (0.99-1.03)	.61	.01	10.93
Per 5-unit increase if >2.5 and ≤45 ng/mL	0.97 (0.95-0.99)	.005		
Per 5-unit increase if >45 ng/mL	1.02 (1.01-1.04)	.002		
White (reference: all other races)^e^	1.19 (1.07-1.32)	.001	NA	10.34
Difficulty crossing the culprit lesion during the PCI procedure	1.30 (1.10-1.54)	.002	NA	9.74
Prior atrial fibrillation or flutter	1.22 (1.05-1.40)	.007	NA	7.19
Age				
Per 5-y increase if ≤65 y	1.03 (1.01-1.06)	.02	.04	6.30
Per 5-y increase if >65 y	0.98 (0.95-1.01)	.12		
Prior heart failure	1.15 (1.01-1.32)	.04	NA	4.19
β Blocker as home medication, yes or no	0.93 (0.85-1.01)	.09	NA	2.97
Left main stenosis ≥50%	1.13 (0.98-1.31)	.09	NA	2.88
Prior MI	1.09 (0.98-1.22)	.12	NA	2.42
Prior PCI	0.93 (0.85-1.03)	.18	NA	1.78
Proximal LAD stenosis ≥70%	1.04 (0.97-1.12)	.24	NA	1.37
Hemoglobin level, per 1-unit decrease in initial value	1.01 (0.99-1.03)	.35	NA	0.89
Cerebrovascular disease	0.95 (0.83-1.08)	.43	NA	0.63
Prior CABG	0.96 (0.82-1.11)	.57	NA	0.33
Peripheral artery disease	1.04 (0.91-1.19)	.59	NA	0.29
Hispanic or Latino ethnicity	0.96 (0.82-1.13)	.62	NA	0.25
Heart rate at admission, per 5-unit increase	1.00 (0.99-1.01)	.66	NA	0.19
Time from first medical contact to PPCI, per 10-min increase	1.00 (0.99-1.01)	.77	NA	0.08

Abbreviations: AOR, adjusted odds ratio; BP, blood pressure; CABG, coronary artery bypass graft; eCrCl, estimated creatinine clearance; LAD, left anterior descending artery; LVEF, left ventricular ejection fraction; MI, myocardial infarction; NA, not applicable; PCI, percutaneous coronary intervention; PPCI, primary percutaneous coronary intervention; VF, ventricular fibrillation; VT, ventricular tachycardia.

SI conversion factor: To convert troponin to μg/L, multiply by 1.0.

^a^ C-index, 0.663.

^b^ For multivariate analysis, variables were sorted by χ^2^ value.

^c^ Cockcroft-Gault formula.

^d^ Weekends or 5 to 8 pm on weekdays.

^e^ Grouped because of small sample sizes.

Model performance improved when examining factors associated with late VT or VF with cardiac arrest (C-index, 0.826). Factors associated with increased odds of late VT or VF and cardiac arrest are presented in Table 3. The most significant factors were decreasing LVEF (AOR for every 5-unit decrease ≤40%: 1.67; 95% CI, 1.54-1.85), difficulty crossing the culprit lesion during PCI (AOR, 1.60; 95% CI, 1.16-2.20), heart failure at admission (AOR, 1.53; 95% CI, 1.24-1.91), cardiogenic shock at admission (AOR, 1.42; 95% CI, 1.10-1.85), and 3-vessel coronary artery disease (AOR, 1.57; 95% CI, 1.28-1.92). Given the low rates of cardiac arrest with late VT or VF in the low-risk cohort, a separate model was not developed in this group.

**Table 3.** Factors Associated With Risk of Late VT or VF With Cardiac Arrest in the Overall Cohort

Factor	AOR (95% CI)^a^	Individual *P* value	Global *P* value	χ^2^^b^
LVEF				
Per 5-unit decrease if ≤40%	1.67 (1.54-1.85)	<.001	<.001	130.18
Per 5-unit decrease if >40%	1.21 (1.14-1.32)	<.001		
Initial troponin level × upper limit value, per 5-unit increase	1.04 (1.03-1.05)	<.001	NA	60.62
Systolic BP at admission, per 5-unit decrease	1.04 (1.03-1.06)	<.001	NA	34.06
Initial eCrCl^c^				
Per 5-unit decrease if ≤110 mL/min	1.05 (1.03-1.08)	<.001	<.001	22.32
Per 5-unit decrease if >110 mL/min	0.96 (0.93-0.99)	.01		
Diseased vessels, No.				
2 vs ≤1	1.30 (1.08-1.57)	.006	<.001	18.60
3 vs ≤1	1.57 (1.28-1.92)	<.001		
Age				
Per 5-y increase if ≤60 y	1.21 (1.09-1.35)	<.001	<.001	15.02
Per 5-y increase if >60 y	1.00 (0.95-1.06)	.97		
Heart failure at admission	1.53 (1.24-1.91)	<.001	NA	15.01
Heart rate at admission				
Per 5-unit increase if ≤80 bpm	0.96 (0.91-1.01)	.10	<.001	14.31
Per 5-unit increase if >80 bpm	1.08 (1.04-1.12)	<.001		
Difficulty crossing the culprit lesion during the PCI procedure	1.60 (1.16-2.20)	.004	NA	8.37
Prior CABG	0.61 (0.44-0.86)	.004	NA	8.09
Cardiogenic shock at admission	1.42 (1.10-1.85)	.008	NA	7.10
Diabetes	1.24 (1.05-1.47)	.01	NA	6.38
Left main stenosis ≥50%	1.40 (1.06-1.84)	.02	NA	5.65
Time from first medical contact to PPCI, per 10-min increase	1.02 (1.00-1.04)	.05	NA	3.77
Female (reference: male)	0.86 (0.73-1.01)	.07	NA	3.26
Prior atrial fibrillation or flutter	1.27 (0.98-1.66)	.07	NA	3.25
Off-hours presentation^d^	1.16 (0.99-1.36)	.08	NA	3.17
β Blocker as home medication, yes vs no	1.17 (0.97-1.42)	.09	NA	2.86
Hemoglobin level, per 1-unit decrease in initial value	1.04 (0.99-1.10)	.10	NA	2.77
Proximal LAD stenosis ≥70%	1.12 (0.95-1.31)	.16	NA	1.94
White (reference: all other races)^e^	1.13 (0.88-1.46)	.33	NA	0.95
Prior heart failure	1.15 (0.87-1.53)	.33	NA	0.93
Prior PCI	0.91 (0.70-1.18)	.48	NA	0.51
Cerebrovascular disease	1.09 (0.85-1.39)	.52	NA	0.42
Prior MI	1.06 (0.81-1.40)	.66	NA	0.20
Peripheral artery disease	1.05 (0.78-1.42)	.74	NA	0.11
Hispanic or Latino ethnicity	0.98 (0.66-1.46)	.93	NA	0.01

Abbreviations: AOR, adjusted odds ratio; BP, blood pressure; CABG, coronary artery bypass graft; eCrCl, X creatinine clearance; LAD, left anterior descending artery; LVEF, left ventricular ejection fraction; MI, myocardial infarction; NA, not applicable; PCI, percutaneous coronary intervention; PPCI, primary percutaneous coronary intervention; VF, ventricular fibrillation; VT, ventricular tachycardia.

^a^ C-index, 0.826.

^b^ For multivariate analysis, variables were sorted by χ^2^ value.

^c^ Cockcroft-Gault formula.

^d^ Weekends or 5 to 8 pm on weekdays.

^e^ Grouped because of small sample sizes.

### In-Hospital Events Associated With Late VT or VF

Unadjusted and adjusted odds of in-hospital mortality were higher in patients with late VT or VF compared with those without VT or VF. In the overall cohort, the AOR was 6.40 (95% CI, 5.63-7.29; *P* < .001) compared with 8.74 (95% CI, 6.53-11.70; *P* < .001) in the cohort with uncomplicated STEMI (eTable 2 in Supplement 1).

## Discussion

In this cohort study of a contemporary, national population with STEMI treated with primary PCI, we (1) measured the risk and timing of late VT or VF, (2) identified factors associated with late VT or VF, and (3) measured the in-hospital mortality associated with late VT or VF. Important findings from this study include (1) the risk of any VT or VF after primary PCI for STEMI was 8.9% in the overall population and 5.4% in the subpopulation with uncomplicated STEMI; (2) the risk of late VT or VF events (occurring ≥1 day after PCI) was 2.4% in the overall population and 1.7% in a cohort with uncomplicated STEMI; (3) late VT or VF associated with cardiac arrest was uncommon and occurred in only 0.4% of the overall population and 0.1% of the cohort with uncomplicated STEMI; (4) the ability to estimate any late VT or VF events was fair, but the ability to estimate late VT or VF events associated with cardiac arrest was good (C-index, 0.826); (5) multiple patient and presenting factors were associated with late VT or VF events, with reduced LVEF as the most significant factor; and (6) the odds of experiencing in-hospital death were significantly higher in patients with late VT or VF events.

This study found a risk of overall VT or VF that was consistent with previous reports in populations with an MI^1,2,5^ despite advances in primary PCI and systems of care for patients with STEMI. However, this finding may be related to the definition of VT or VF in the NCDR Chest Pain–MI Registry as encompassing any run of 7 or more beats of ventricular tachycardia or fibrillation. While this definition may have led to a higher risk of VT or VF in our study, this study represents a clinical evaluation of VT or VF after primary PCI for reperfusion of STEMI. Additionally, prior studies included secondary analyses of trials,^2,4^ which may include more restrictive populations than the general population with STEMI in the US. This may explain the lower rates of VT or VF reported in past publications that are more consistent with the risk of VT or VF observed in the cohort with uncomplicated STEMI in the present analysis.

Late VT or VF events after STEMI remain a concern, with current practice patterns typically including at least 2 days of in-hospital telemetry heart rhythm monitoring regardless of a patient’s risk profile after STEMI. Our study found a risk of late VT or VF events of 2.4% in the overall population and 1.7% in the cohort with uncomplicated STEMI. More importantly, late VT or VF associated with cardiac arrest on the same day of the VT or VF occurred infrequently, with only 0.4% of the overall cohort and 0.1% of patients with uncomplicated STEMI experiencing events. Although these event rates are low, when present, they were associated with a higher risk of in-hospital mortality in the overall and low-risk cohorts. As STEMI management has become increasingly routine, with many patients no longer admitted to an intensive care unit after reperfusion,^14,15^ questions remain regarding the optimal duration of in-hospital telemetry monitoring. In cases for which early discharge is being considered, the risks of late VT or VF and cardiac arrest should be discussed with patients to ensure appropriate shared decision-making regarding the timing of discharge.

Identifying predictors of late VT or VF is paramount to identifying high-risk patients who should have longer in-hospital monitoring while also facilitating safe, early discharge for low-risk individuals after successful reperfusion. Our study showed that identifying factors associated with any late VT or VF is challenging (C-index, 0.663), but identifying factors associated with late VT or VF and cardiac arrest can be achieved with higher accuracy (C-index, 0.826). Factors associated with late VT or VF with cardiac arrest in our study included reduced LVEF, heart failure or cardiogenic shock at admission, lower blood pressure, extremes of heart rate at presentation, increased number of diseased vessels on angiogram at presentation, worsening kidney function, and difficulty crossing the culprit lesion. Interestingly, increasing time from first medical contact to PCI was not significantly associated with late VT or VF with cardiac arrest after adjustment. Limited data exist examining factors associated with late VT or VF after STEMI, and our findings support prior reports^2,6,8^ of risk factors for ventricular arrhythmias after STEMI within the context of a contemporary clinical population with STEMI treated with primary PCI. To our knowledge, this study represents the largest population examined to date (n = 174 126) and encompasses a contemporary population with STEMI treated by timely primary PCI.

As expected, our study found that late VT or VF was associated with a significantly increased risk of mortality for patients with late VT or VF in both the overall cohort and the cohort with uncomplicated STEMI. Similar findings have been noted in other acute MI studies.^2,6,7,8^ Although late VT or VF events are relatively infrequent, they can have devastating consequences for patients when they do occur. This reinforces the need for additional factors that may predict safe, early discharge following STEMI with adequate revascularization and novel approaches to out-of-hospital monitoring for ventricular arrhythmias following MI. Current guidelines^11,12^ lack clear guidance on the optimal duration of telemetry monitoring and recommendations for early discharge following primary PCI for uncomplicated STEMI. While decisions regarding the timing of safe discharge for a patient should always be tailored to an individual, this study provides important information to customize an informed, shared decision-making conversation with a patient about their risk of a dangerous arrhythmia following STEMI treated with PCI in the modern era.

### Limitations

This study should be evaluated within the context of its limitations. First, exact time stamps are not available for post-PCI events. As such, we approximated the time from reperfusion to an event based on the day on which it occurred. This may have led to variation in the time to event based on the time of day when the primary PCI and subsequent VT or VF event occurred. Second, as this was an observational study of registry data, event definitions were predefined within the registry data dictionary. The definition of VT included any series of 7 or more consecutive ventricular complexes. As such, it is difficult to determine whether these VT episodes were sustained or hemodynamically significant. Using the Chest Pain–MI Registry, we were unable to separate VT and VF occurrences. We recognize this limitation, as VF and VT have different prognoses. The AOR for mortality was higher in late VT or VF for the cohort with uncomplicated STEMI compared with the overall cohort. As there are substantial differences in sample sizes between these cohorts, this AOR difference may be due to sample size differences. Additionally, as this analysis was limited to in-hospital data, any late VT or VF events occurring after discharge were not captured. Moreover, we could not capture whether patients had undergone complete revascularization (of the nonculprit lesions) at the time of a VT or VF episode. Finally, despite the use of logistic regression modeling, it is possible that unmeasured confounding or bias may have influenced our results.

## Conclusions

In this cohort study, late VT or VF occurring at least 1 day after primary PCI was seen in 2.4% of patients with STEMI undergoing primary PCI. Late VT or VF associated with cardiac arrest was rare, particularly in lower-risk patients with uncomplicated STEMI. Several factors were associated with late VT or VF and cardiac arrest, with decreasing LVEF being the most significant factor associated with events. In both the overall cohort and in patients with uncomplicated STEMI, late VT or VF was associated with increased in-hospital mortality. This underscores the importance of close patient monitoring of patients with STEMI in the early days after primary PCI, while also providing useful information for shared decision-making about earlier discharge in low-risk individuals.

## Notes

Supplement 1. **eTable 1.** Baseline Characteristics of Patients With and Without Late VT or VF in the Cohort With Uncomplicated STEMI **eTable 2.** Unadjusted and Adjusted Odds of In-Hospital Mortality Associated With Late VT or VF Events **eFigure 1.** Flow Diagram **eFigure 2.** Occurrence of VT or VF Overall and in the Low-Risk Cohort

Supplement 2.
Data Sharing Statement
