# Supplementary material for: Ventricular Arrhythmias After Primary Percutaneous Coronary Intervention for STEMI
Source: JAMA Netw Open. 2024 May 8;7(5):e2410288. doi: 10.1001/jamanetworkopen.2024.10288 (PMC11079687; doi:10.1001/jamanetworkopen.2024.10288)
Supplement: Supplement 1. — eTable 1. Baseline Characteristics of Patients With and Without Late VT or VF in the Cohort With Uncomplicated STEMI eTable 2. Unadjusted and Adjusted Odds of In-Hospital Mortality Associated With Late VT or VF Events eFigure 1. Flow Diagram eFigure 2. Occurrence of VT or VF Overall and in the Low-Risk Cohort [file jamanetwopen-e2410288-s001.pdf]

Supplementary Online Content

Rymer JA, Wegermann ZK, Wang TY, et al. Ventricular arrhythmias after primary percutaneous coronary intervention for STEMI. *JAMA Netw Open*. 2024;7(5):e2410288. doi:10.1001/jamanetworkopen.2024.10288

eTable 1. Baseline Characteristics of Patients With and Without Late VT or VF in the Cohort With Uncomplicated STEMI

eTable 2. Unadjusted and Adjusted Odds of In-Hospital Mortality Associated With Late VT or VF Events

eFigure 1. Flow Diagram

eFigure 2. Occurrence of VT or VF Overall and in the Low-Risk Cohort

This supplementary material has been provided by the authors to give readers additional information about their work.

© 2024 Rymer JA et al. *JAMA Network Open*.

eTable 1. Baseline Characteristics of Patients With and Without Late VT/VF in the Uncomplicated STEMI Cohort

Variable (%)	Overall (N=96188) N (%)	No VT/VF (N=94473) N (%)	Late VT/VF (N=1715) N (%)	*P*-value
**Demographics**				
Age, years*	60 (52-69)	60 (52-69)	60 (53-69)	0.0304
Weight, Kg*	86.20 (74.84-100.00)	86.20 (74.84-100.00)	87.00 (76.50-99.79)	0.4525
Body mass index, kg/m2*	28.88 (25.69-32.82)	28.89 (25.70-32.82)	28.49 (25.36-32.24)	0.0065
Race				<.0001
White	77477 (80.55)	76017 (80.46)	1460 (85.13)	
Black	8452 (8.79)	8326 (8.81)	126 (7.35)	
Asian	2438 (2.53)	2413 (2.55)	25 (1.46)	
Hispanic	6537 (6.80)	6453 (6.83)	84 (4.90)	
Other	547 (0.57)	539 (0.57)	28 (0.47)	
**Medical history**				
Current/recent smoker (<1 year)	37805 (39.30)	37103 (39.27)	702 (40.93)	0.1654
Hypertension	58395 (60.71)	57455 (60.82)	940 (54.81)	<0.0001
Dyslipidemia	45118 (46.91)	44361 (46.96)	757 (44.14)	0.0200
Diabetes mellitus	22953 (23.86)	22645 (23.97)	308 (17.96)	<0.0001
Prior PCI	6239 (6.49)	6138 (6.50)	101 (5.89)	0.3095
Prior CABG	2307 (2.40)	2251 (2.38)	56 (3.27)	0.0180
Atrial fibrillation or flutter	3017 (3.14)	2945 (3.12)	72 (4.20)	0.0111
Cerebrovascular disease	4715 (4.90)	4651 (4.92)	64 (3.73)	0.0234
Peripheral arterial disease	2642 (2.75)	2598 (2.75)	44 (2.57)	0.6444
On dialysis	417 (0.43)	413 (0.44)	4 (0.23)	0.2024
Cancer	6837 (7.11)	6678 (7.07)	159 (9.27)	0.0005
**Home Medications**				
Aspirin	22351 (23.24)	21997 (23.28)	354 (20.64)	0.0098
P2Y12 Inhibitors	3367 (3.50)	3324 (3.52)	43 (2.51)	0.0238
ACE/ARB	25259 (26.26)	24854 (26.31)	405 (23.62)	0.0118
Beta Blocker	15945 (16.58)	15693 (16.61)	252 (14.69)	0.0340
Statin	22475 (23.37)	220947(23.39)	381 (22.22)	0.2492

© 2024 Rymer JA et al. *JAMA Network Open*.

eTable 1. Baseline Characteristics of Patients With and Without Late VT/VF in the Uncomplicated STEMI Cohort

Variable (%)	Overall (N=96188) N (%)	No VT/VF (N=94473) N (%)	Late VT/VF (N=1715) N (%)	*P*-value
**Signs and symptoms at presentation**				
Location patient first evaluated:				0.1674
Emergency department	70845 (73.65)	69581 (73.65)	1264 (73.70)	
Catheterization laboratory	23898 (24.85)	23464 (24.84)	434 (25.31)	
Other	1345 (1.40)	1330 (1.41)	15 (0.87)	
First ECG obtained:				0.0004
Pre-hospital	38998 (40.54)	38231 (40.47)	767 (44.72)	
After first hospital arrival	57096 (59.36)	56148 (59.43)	948 (53.28)	
Mean of transport to first facility:				<0.0001
Self/family	51783 (53.84)	50931 (53.91)	852 (49.68)	
Ambulance	42973 (44.68)	42154 (44.62)	819 (47.76)	
Air	1351 (1.40)	1307 (1.38)	44 (2.57)	
Onset to arrival, hours*	1.58 (0.97-3.17)	1.58 (0.97-3.17)	1.68 (1.03-3.03)	0.0186
Arrival to Cath, minutes	0.73 (0.50-1.02)	0.73 (0.50-1.02)	0.72 (0.48-0.97)	0.0050
Heart rate on admission, bpm*	77 (65-90)	77 (65-90)	77 (65-88)	0.3359
Systolic BP on admission, mmHg*	150 (130-170)	150 (130-170)	148 (129-167)	0.0057
**Laboratory Results and Diagnostic Data**				
Number of Diseased vessels				<0.0001
0	607 (0.63)	601 (0.64)	6 (0.35)	
1	46373 (48.48)	45617 (48.56)	756 (44.13)	
2	29148 (30.47)	28625 (30.47)	523 (30.53)	
3	19452 (20.33)	19025 (20.25)	427 (24.93)	
LV Ejection Fraction (%)				<0.0001
>50	67697 (70.38)	66651 (70.55)	1046 (60.99)	
40-50	28491 (29.62)	27822 (29.45)	669 (39.01)	

© 2024 Rymer JA et al. *JAMA Network Open*.

eTable 1. Baseline Characteristics of Patients With and Without Late VT/VF in the Uncomplicated STEMI Cohort

Variable (%)	Overall (N=96188) N (%)	No VT/VF (N=94473) N (%)	Late VT/VF (N=1715) N (%)	*P*-value
eGFR initial (non-dialysis)*				0.0149
<30	1531 (1.60)	1502 (1.60)	29 (1.69)	
30-<60	13245 (13.83)	12992 (13.81)	253 (14.79)	
60-<90	29725 (31.04)	29142 (30.98)	583 (34.07)	
≥90	50527 (52.76)	19685 (52.82)	842 (49.21)	
Hemoglobin initial, g/dL*	14.80 (13.60-15.90)	14.8 (13.60-15.90)	14.9 (13.75-15.90)	0.0576
Troponin initial, X upper limit value*	2.00 (0.40-23.33)	2.00 (0.40-23.45)	1.67 (0.50-17.20)	0.3714

Abbreviations: BP, blood pressure; CABG, coronary artery bypass graft; ECG, electrocardiogram; eGFR, estimated glomerular filtration rate; LV, left ventricular; PCI, percutaneous coronary intervention; VF, ventricular fibrillation; VT, ventricular tachycardia.

[a]Median and interquartile ranges are reported.

© 2024 Rymer JA et al. *JAMA Network Open*.

eTable 2. Unadjusted and Adjusted Odds of In-hospital Mortality Associated With Late VT/VF Events

Population	Unadjusted OR (95% CI)	Unadjusted *P*-value	Adjusted OR (95% CI)	Adjusted *P*-value
Overall Cohort	7.29 (6.51 - 8.16)	<.001	6.40 (5.63 - 7.29)	<.001
Uncomplicated STEMI Cohort	8.55 (6.47 - 11.29)	<.001	8.74 (6.53 - 11.70)	<.001

CI, confidence interval; STEMI, ST Elevation Myocardial Infarction; VF, ventricular fibrillation; VT, ventricular tachycardia.

© 2024 Rymer JA et al. *JAMA Network Open*.

eFigure 1. Flow Diagram

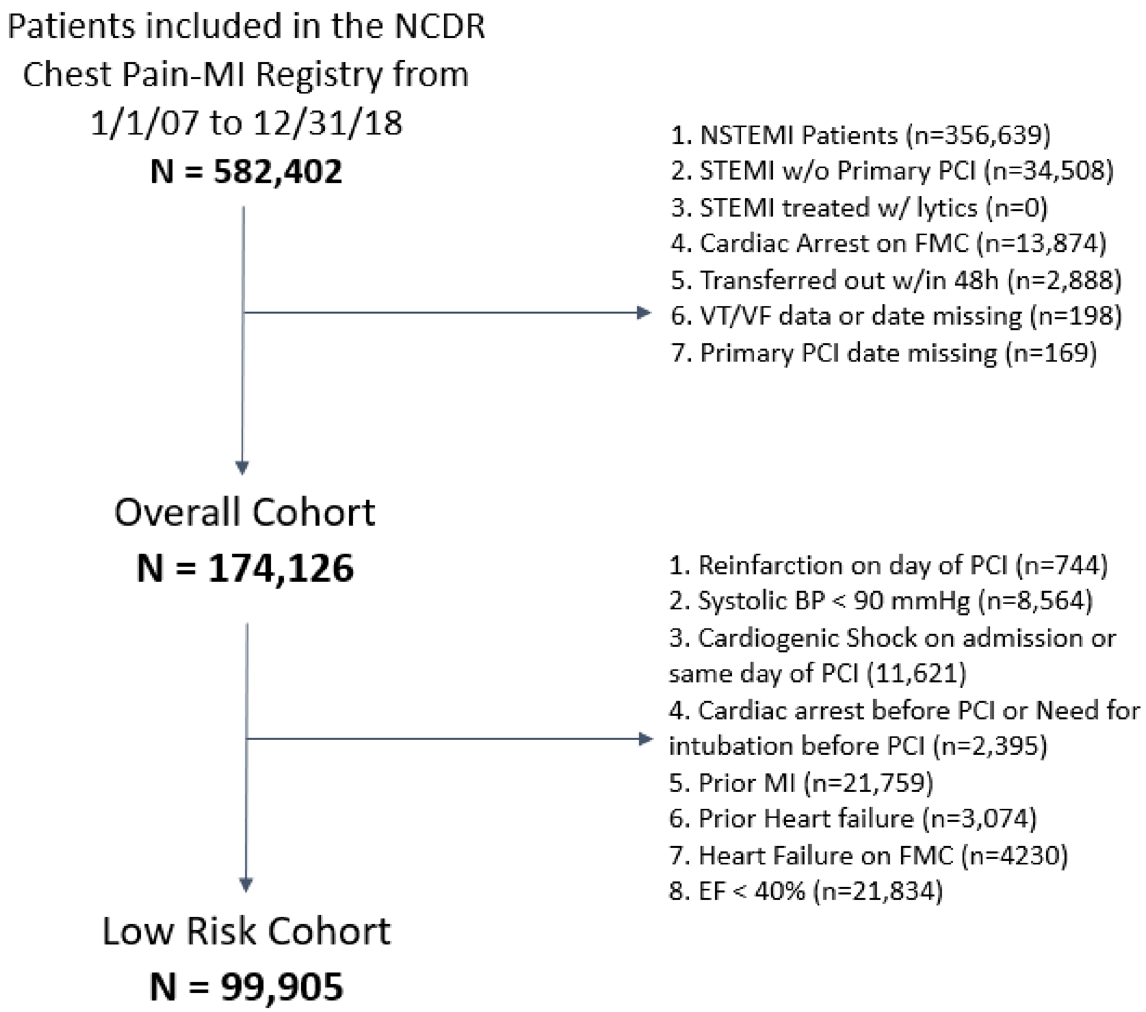

The figure depicts the process used to acquire the final sample size of patients including in the current analysis. The number and reason for exclusion of patient records is depicted in the side boxes and explained in the methodology. Abbreviations: BP, blood pressure; EF, ejection fraction; FMC, first medical contract; MI, myocardial infarction; NCDR, National Cardiovascular Data Registry; NSTEMI, non ST elevation infarction; PCI, percutaneous coronary intervention; STEMI, ST elevation infarction; VT/VF, ventricular tachycardia/ventricular fibrillation; w/-, with; w/o, without.

© 2024 Rymer JA et al. *JAMA Network Open*.

eFigure 2. Occurrence of VT/VF Overall and in the Low-risk Cohort

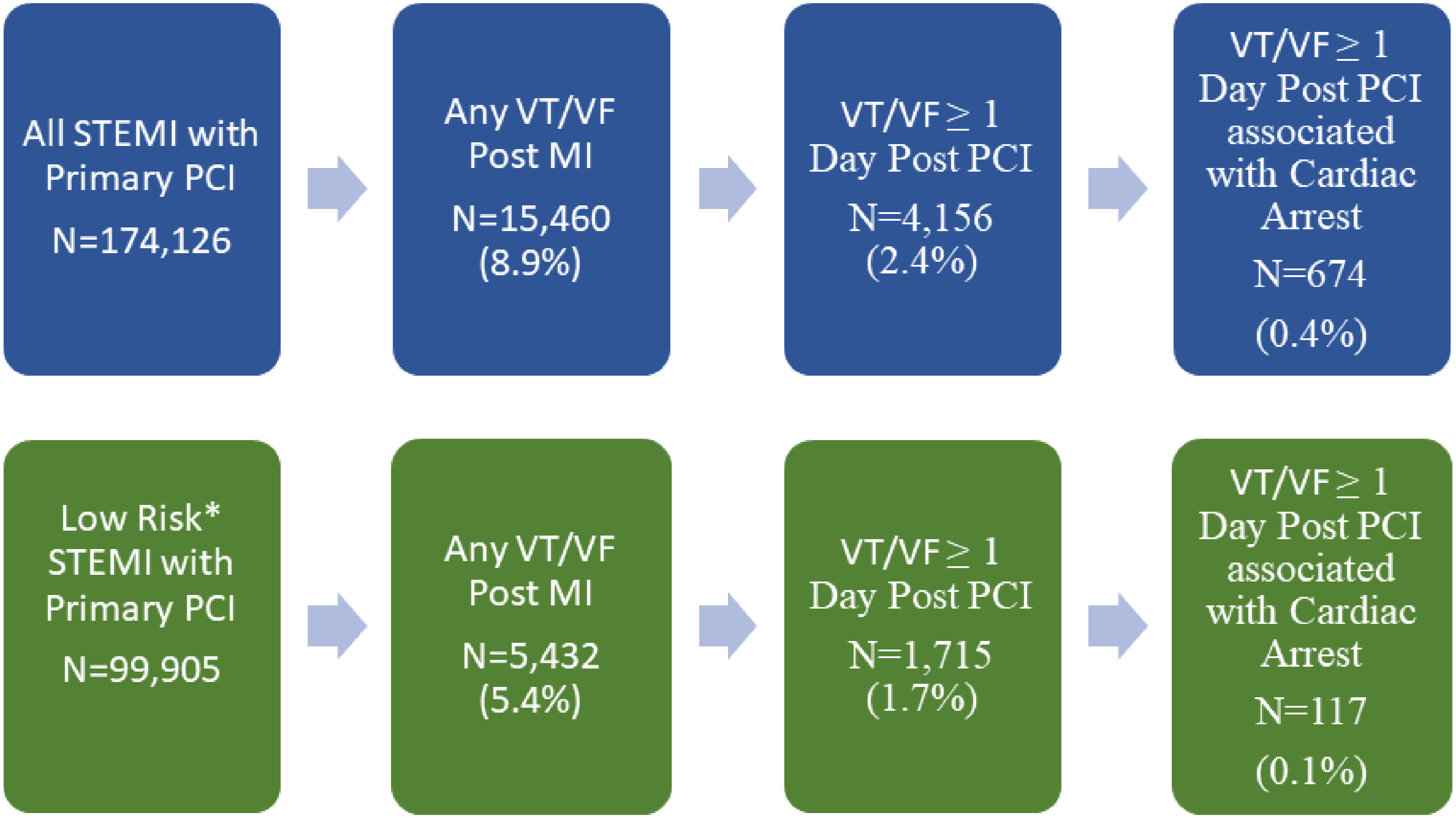

The figure depicts the proportion of patients with any VT/VF, Late VT/VF, and Late VT/VF associated with cardiac arrest in the overall population and the low-risk cohort. *Low Risk defined by the absence of prior MI, hear failure, systolic blood pressure <90 mmHg, cardiogenic shock, cardiac arrest, re-infarction, or ejection fraction <40%. Abbreviations: MI, myocardial infarction; PCI, percutaneous coronary intervention; STEMI, ST elevation infarction; VT/VF, ventricular tachycardia/ventricular fibrillation.

© 2024 Rymer JA et al. *JAMA Network Open*.
