# Supplementary material for: Ventricular Arrhythmias After Primary Percutaneous Coronary Intervention for STEMI
Source: JAMA Netw Open. 2024 May 8;7(5):e2410288. doi: 10.1001/jamanetworkopen.2024.10288 (PMC11079687; doi:10.1001/jamanetworkopen.2024.10288)
Supplement: Supplement 2. — Data Sharing Statement [file jamanetwopen-e2410288-s002.pdf]

Data Sharing Statement

Rymer. Ventricular Arrhythmias After Primary Percutaneous Coronary Intervention for STEMI. *JAMA Netw Open*. Published May 08, 2024. doi:10.1001/jamanetworkopen.2024.10288

Data
Data available: No

Additional Information
Explanation for why data not available: Data collected from NCDR registry. Authors do not have direct access to this data
